# Efficacy of Gegen Qinlian decoction plus metformin for type 2 diabetes mellitus: a systematic review and meta-analysis of randomized controlled trials

**DOI:** 10.3389/fendo.2026.1837588

**Published:** 2026-07-17

**Authors:** Hongyang He, Xiaocan Gai, Zhiheng Qiao, Guoliang Ji, Shumin Liu

**Affiliations:** 1 Graduate School, Heilongjiang University of Chinese Medicine, Harbin, China; 2 School of Basic Medical Sciences, Heilongjiang University of Chinese Medicine, Harbin, China

**Keywords:** Gegen Qinlian decoction, meta-analysis, metformin, randomized controlled trials, type 2 diabetes mellitus

## Abstract

**Background:**

Gegen Qinlian Decoction (GQD) is frequently used as an adjunctive therapy to metformin for type 2 diabetes mellitus (T2DM), but the certainty and consistency of the supporting clinical evidence remain unclear.

**Methods:**

We searched CNKI, Wanfang Data, PubMed, EMBASE, Web of Science, and the Cochrane Central Register of Controlled Trials from database inception to January 31, 2026. Randomized controlled trials comparing GQD plus metformin with metformin alone in adults with T2DM were included. The primary outcome was glycated hemoglobin (HbA1c), and secondary outcomes were fasting plasma glucose (FPG) and 2-hour postprandial glucose (2hPG). Risk of bias was assessed using the Cochrane RoB 2 tool, and the certainty of evidence was evaluated using the GRADE framework. Random-effects meta-analyses were performed, with the primary analyses based on change-from-baseline values.

**Results:**

Eight randomized controlled trials involving 725 participants were included. In the primary change-score analysis, GQD plus metformin did not show a statistically significant reduction in HbA1c compared with metformin alone (mean difference [MD] = -1.92, 95% confidence interval [CI]: -4.43 to 0.59, P = 0.13). Statistically significant reductions were observed for FPG (MD = -1.08, 95% CI: -1.71 to -0.44, P = 0.0009) and 2hPG (MD = -1.73, 95% CI: -3.05 to -0.42, P = 0.010). However, substantial heterogeneity was present across analyses (I² = 85%–100%), and the certainty of evidence was rated as very low because of risk of bias, inconsistency, and imprecision. Post-treatment sensitivity analyses using all eight trials showed statistically significant reductions in HbA1c, FPG, and 2hPG, but these analyses were also affected by substantial heterogeneity and high risk of bias across the included trials.

**Conclusion:**

Current evidence is insufficient to confirm a reliable clinical benefit of GQD as an adjunct to metformin for T2DM. Although reductions in FPG and 2hPG were observed, these findings should be interpreted as exploratory because of substantial heterogeneity, methodological limitations, and very low certainty of evidence. Larger, prospectively registered, well-designed, and adequately blinded randomized trials are needed.

## Introduction

Type 2 diabetes mellitus (T2DM) is a chronic metabolic disease characterized by hyperglycemia resulting from insulin resistance and progressive β-cell dysfunction (1). Its prevalence continues to increase worldwide, creating a substantial burden for patients and healthcare systems (2). Metformin remains a widely recommended first-line pharmacological treatment because of its established glucose-lowering efficacy, safety profile, low cost, and broad clinical experience (3). However, glycemic control may become insufficient over time in some patients receiving metformin monotherapy, and additional therapeutic strategies are often required to achieve or maintain individualized glycemic targets (3).

Gegen Qinlian Decoction (GQD), also known as Ge Gen Qin Lian Tang, is a classical traditional Chinese medicine formula that is used in China as an adjunctive treatment for metabolic and gastrointestinal disorders, including T2DM-related symptoms (4). In clinical practice, GQD is sometimes combined with metformin with the aim of improving glycemic control. Several randomized trials have evaluated GQD plus metformin for T2DM, and recent studies have also explored potential mechanisms involving gut microbiota and metabolic regulation (5). However, the clinical evidence remains uncertain because many available trials are small, have limited methodological reporting, and differ in treatment duration, intervention details, and outcome reporting.

Therefore, we conducted this systematic review and meta-analysis to evaluate the efficacy and certainty of evidence for GQD plus metformin compared with metformin alone in adults with T2DM. To maintain a focused clinical question, we included only randomized controlled trials directly comparing these two treatment strategies and excluded studies that used additional hypoglycemic drugs. The primary outcome was HbA1c, and the secondary outcomes were FPG and 2hPG.

## Methods

### Study design and reporting guideline

This systematic review and meta-analysis was conducted and reported in accordance with the PRISMA 2020 statement (6). The completed PRISMA 2020 checklist is provided in **Appendix S1**. Detailed search strategies for all electronic databases are provided in **Appendix S2**. Detailed information on the GQD regimens and metformin doses used in the included trials is provided in **Appendix S3**.

### Eligibility criteria

Studies were selected according to predefined PICOS criteria. The population was adults diagnosed with type 2 diabetes mellitus. The intervention was GQD combined with metformin, and the comparator was metformin alone. The eligible outcomes were quantitative glycemic outcomes, including HbA1c, FPG, and 2hPG. The eligible study design was randomized controlled trials, regardless of whether the method of random sequence generation was fully described.

To isolate the adjunctive effect of GQD, studies were excluded if the intervention or comparator included additional hypoglycemic drugs other than metformin. Duplicate publications, non-randomized studies, animal or mechanistic studies without eligible clinical outcomes, reviews, case reports, and studies with unavailable or insufficient quantitative outcome data were also excluded.

### Search strategy and data sources

A comprehensive search was conducted across Chinese- and English-language databases, including PubMed, EMBASE, Web of Science, China National Knowledge Infrastructure (CNKI), Wanfang Data, and the Cochrane Central Register of Controlled Trials. All databases were searched from inception to January 31, 2026. The search strategy focused on three main concepts: GQD, type 2 diabetes, and randomized controlled trials. Detailed search strategies are presented in **Appendix S2**.

### Study selection and data extraction

Two reviewers independently screened titles, abstracts, and full texts according to the predefined eligibility criteria. Disagreements were resolved through discussion or consultation with a third reviewer. For potentially eligible studies for which full texts could not be accessed, attempts were made to obtain the full text through database links, institutional access, and citation tracing.

Two reviewers independently extracted data from the included studies. Extracted information included first author, publication year, country or region, sample size, intervention and comparator details, GQD regimen, metformin dose, treatment duration, randomization method, reported outcomes, baseline values, post-treatment values, SDs, and adverse-event information when available. For continuous glycemic outcomes, means and SDs were extracted for baseline and post-treatment values whenever reported. When multiple post-treatment time points were available, the final assessment at the end of the intervention period was used.

### Outcomes

The primary outcome was HbA1c (%). The secondary outcomes were FPG (mmol/L) and 2hPG (mmol/L). Safety outcomes, including adverse events and hypoglycemic episodes, were extracted when reported; however, they were analyzed descriptively because reporting was insufficient and inconsistent across trials.

### Risk of bias assessment

The methodological quality of the included randomized controlled trials was assessed using the Cochrane Risk of Bias 2 (RoB 2) tool (7). Two reviewers independently assessed each study across the following five domains: bias arising from the randomization process, bias due to deviations from intended interventions, bias due to missing outcome data, bias in measurement of the outcome, and bias in selection of the reported result. Each domain was judged as “low risk of bias,” “some concerns,” or “high risk of bias” according to the RoB 2 guidance.

**Figure 1:**
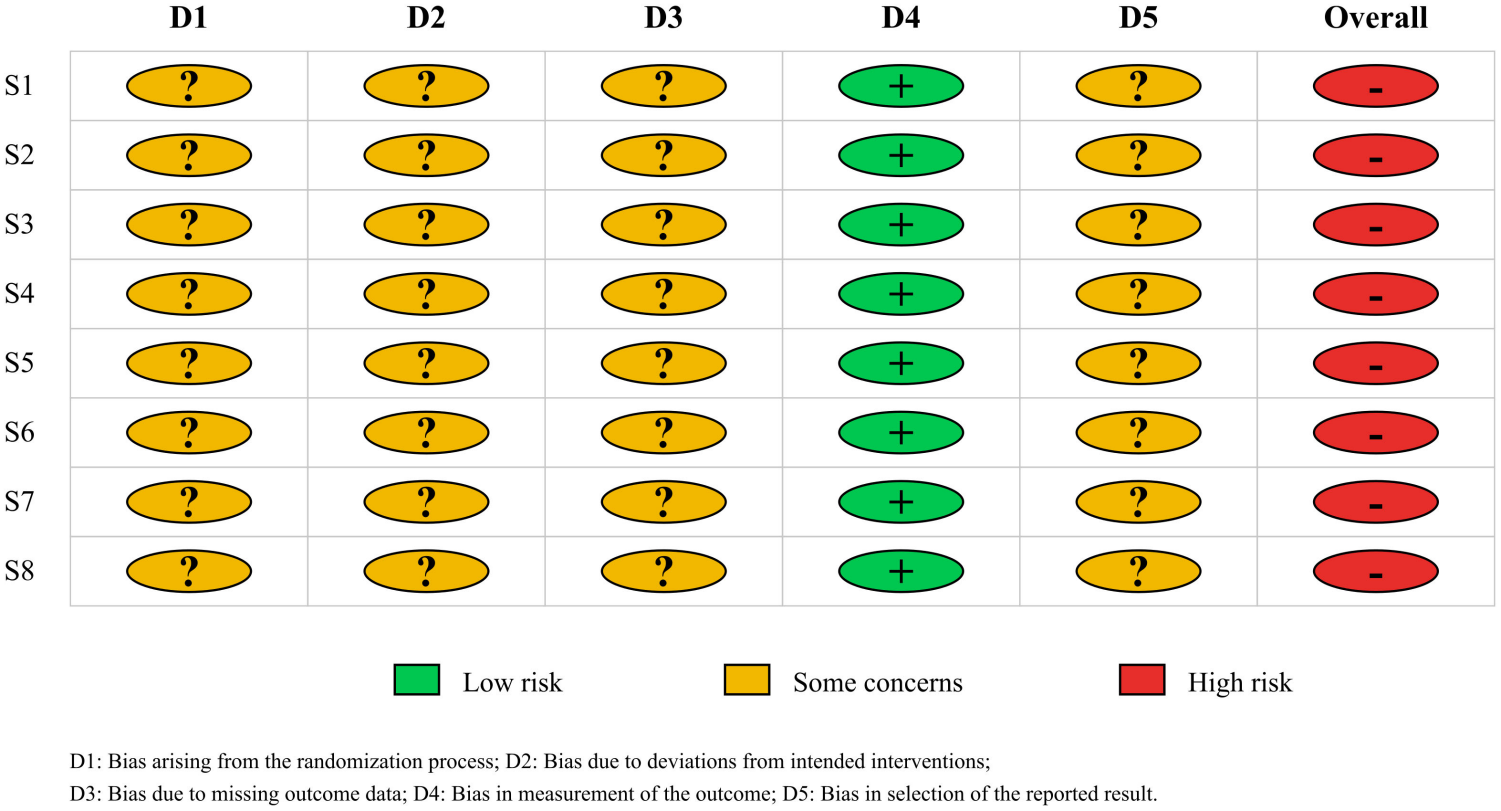
RoB 2 traffic-light plot. Assessment of the risk of bias for each included study (S1–S8) using the Cochrane RoB 2 tool across five domains: randomization process, deviations from intended interventions, missing outcome data, measurement of the outcome, and selection of the reported result. RoB 2, Cochrane risk-of-bias tool for randomized trials.

**Figure 2:**
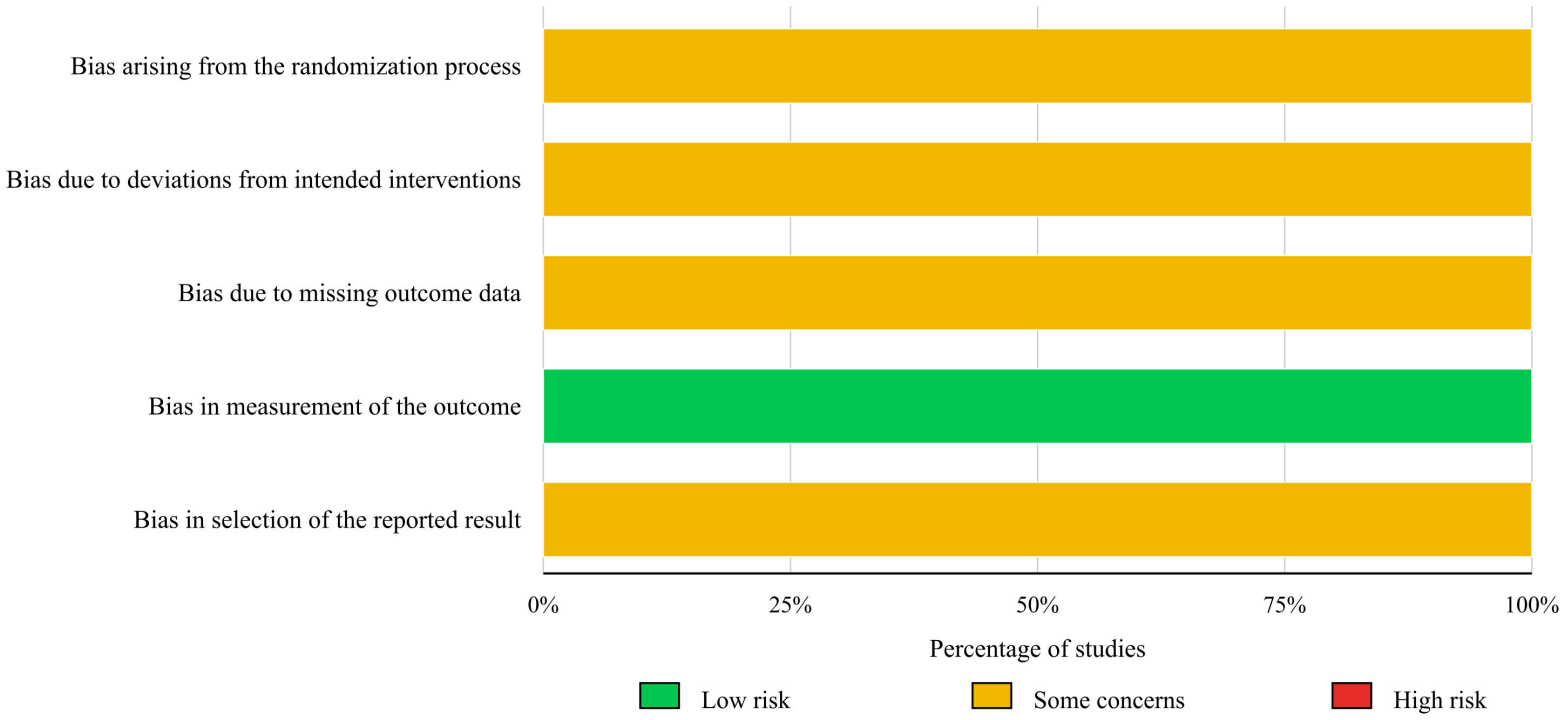
RoB 2 summary plot. Distribution of the risk of bias (low risk, some concerns, and high risk) across all included studies for each of the five RoB 2 domains. RoB 2, Cochrane risk-of-bias tool for randomized trials.

Disagreements between the two reviewers were resolved through discussion or consultation with a third senior reviewer. The overall risk-of-bias judgment for each study was determined based on the domain-level judgments. A study was rated as having an overall high risk of bias if at least one domain was judged as high risk, or if multiple domains raised some concerns. Domain-specific judgments and supporting reasons are provided in **Appendix S4**, and the overall results are illustrated in **Figures 1** and **2**.

### Certainty of evidence assessment

The certainty of evidence for each main outcome was assessed using the Grading of Recommendations Assessment, Development and Evaluation (GRADE) framework (8). The assessed outcomes included HbA1c, FPG, and 2hPG. Because all included studies were randomized controlled trials, the initial certainty of evidence was considered high and was then downgraded according to five domains: risk of bias, inconsistency, indirectness, imprecision, and publication bias.

Two reviewers independently assessed the certainty of evidence for each outcome, and disagreements were resolved through discussion or consultation with a third reviewer. The final certainty of evidence was categorized as high, moderate, low, or very low. A Summary of Findings table was prepared to present the pooled effect estimates, number of participants and studies, certainty ratings, and main reasons for downgrading. No dedicated GRADE software was used; the judgments were made according to the GRADE framework and documented manually in the Summary of Findings table.

### Data analysis

Data management and statistical analyses were performed using RevMan software (version 5.4.1) (9). Continuous outcomes were pooled as mean differences (MDs) with 95% confidence intervals (CIs). Because clinical and methodological variability was expected across the included trials, random-effects models were used for all meta-analyses. Statistical heterogeneity was assessed using the I² statistic (10).

The primary analyses were based on change-from-baseline values because these estimates better account for potential baseline imbalance between treatment groups. When SDs of change scores were not directly reported, they were derived using the standard formula: SD_change = √(SD_baseline² + SD_final² − 2r × SD_baseline × SD_final). This calculation was performed in accordance with guidance in the Cochrane Handbook for Systematic Reviews of Interventions (10). Because within-participant correlations were not reported in the included studies, a correlation coefficient of r = 0.50 was prespecified as a plausible primary assumption. Sensitivity analyses using r = 0.25 and r = 0.75 were conducted to assess whether the primary change-score findings were robust to lower and higher correlation assumptions.

Two additional sensitivity analyses based on post-treatment final values were conducted. The first was restricted to trials with clearly reported randomization methods (S1–S3). The second included all eight trials to examine whether inclusion of trials with unclear randomization reporting changed the direction or statistical significance of the pooled estimates. These final-value analyses were considered supportive sensitivity analyses and were interpreted cautiously because they may be more susceptible to baseline imbalance than change-score analyses.

We considered subgroup analyses according to treatment duration, GQD regimen, and randomization reporting. However, formal subgroup analyses and meta-regression were not performed because the number of available trials was too small, particularly for the primary change-score analyses. Therefore, potential sources of heterogeneity were explored descriptively and considered in the interpretation of the findings. Because fewer than ten studies were available for each outcome, funnel plots and statistical tests for small-study effects were not considered reliable and were not performed. Potential reporting bias was therefore assessed qualitatively on the basis of trial registration status, protocol availability, publication characteristics, and selective-reporting concerns identified during the RoB 2 assessment.

### Review protocol/registration

This systematic review was not prospectively registered in PROSPERO or any other public registry, and no protocol was publicly available before the review was conducted. The eligibility criteria, outcomes, and statistical methods were specified during the review process before the final meta-analyses were undertaken. However, the absence of prospective registration and a publicly accessible protocol may reduce transparency and increase the potential for selective methodological or outcome-related decisions. This limitation was considered when interpreting the findings.

## Results

### Study selection

The database search identified 420 records in total, including 161 from CNKI, 228 from Wanfang Data, 9 from PubMed, 21 from EMBASE, 1 from Web of Science, and 0 from CENTRAL. After removal of 143 duplicate records, 277 records were screened by title and abstract. Of these, 224 records were excluded because they were reviews, animal studies, case reports, did not involve type 2 diabetes mellitus, or did not meet the predefined PICOS criteria. The PubMed search retrieved nine records, including three records indexed as randomized or clinical trials; however, none met the final eligibility criteria after title and abstract screening because they did not evaluate the predefined direct comparison of GQD plus metformin versus metformin alone.

**Figure 3:**
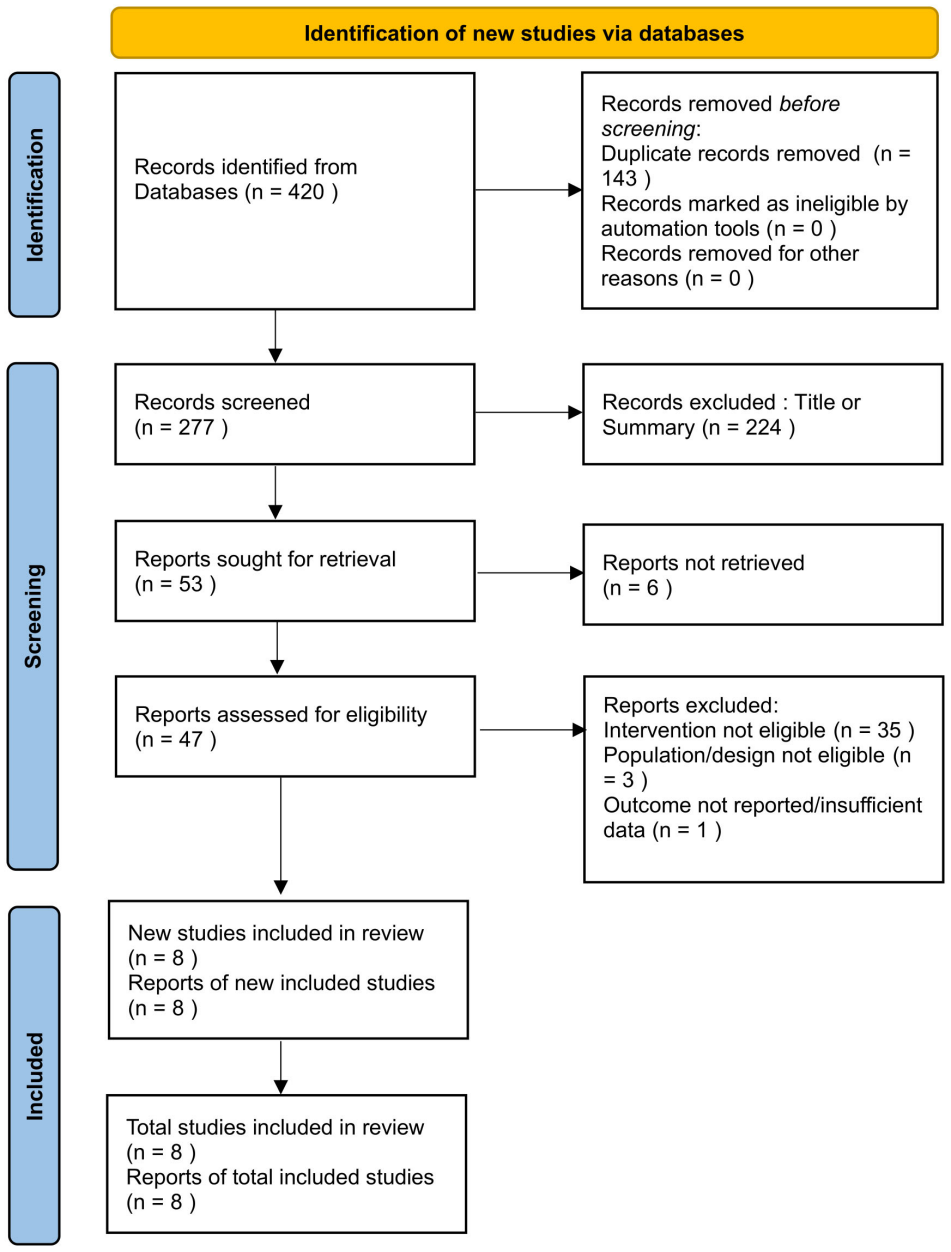
PRISMA flow diagram. The diagram illustrates the study-selection process. A total of 420 records were identified from the databases. After 143 duplicate records were removed, 277 records were screened by title and abstract, and 224 were excluded. Fifty-three reports were sought for full-text retrieval, of which six could not be retrieved. Forty-seven full-text reports were assessed for eligibility, and 39 were excluded because of ineligible intervention (n = 35), ineligible population or study design (n = 3), or unavailable or insufficient outcome data (n = 1). Finally, eight randomized controlled trials were included in the systematic review and meta-analysis.

Fifty-three reports were sought for full-text retrieval. Six reports could not be retrieved through the available database and journal-access routes and were therefore classified as reports not retrieved. The remaining 47 full-text reports were assessed for eligibility. Thirty-nine reports were excluded after full-text assessment for the following reasons: ineligible intervention, mainly because the intervention included additional hypoglycemic drugs or did not evaluate the predefined comparison of GQD plus metformin versus metformin alone (n = 35); ineligible population or study design (n = 3); and unavailable or insufficient quantitative outcome data for meta-analysis (n = 1). Finally, eight randomized controlled trials were included in the systematic review and meta-analysis. The corrected study-selection process is shown in **Figure 3**.

### Study characteristics

The eight included trials enrolled adult patients with type 2 diabetes mellitus and compared GQD plus metformin with metformin alone. The total sample size was 725 participants, with individual study sample sizes ranging from 40 to 169 participants. Treatment duration varied across studies, ranging from 2 weeks to 6 months. All included studies reported at least one glycemic outcome, including HbA1c, FPG, or 2hPG. Detailed information on study characteristics, intervention and control groups, treatment duration, reported outcomes, and randomization methods is presented in **Table 1**.

**Table 1:** Characteristics of the included randomized controlled trials.

Study	Sample size, total (I/C)	Age, years (I/C)	Duration	Randomization method	GQD regimen (dose/frequency)
Li H, 2018 (11)	96 (48/48)	58.10 ± 9.70/59.40 ± 9.80	2 weeks	Random-number table; allocation concealment not reported.	See **Appendix S3** for details
Li WJ and Li M, 2021 (12)	50 (25/25)	55.54 ± 3.45/56.21 ± 4.11	8 weeks	Random-number table; allocation concealment not reported.	See **Appendix S3** for details
Xiong QJ, 2019 (13)	100 (50/50)	53.65 ± 7.65/53.50 ± 7.80	8 weeks	Random-number table; allocation concealment not reported.	See **Appendix S3** for details
Zhang HF, 2019 (14)	70 (35/35)	NR	2 months	Random allocation reported; sequence generation and allocation concealment not described.	See **Appendix S3** for details
Pang XY et al., 2018 (15)	90 (45/45)	53.50 ± 8.20/54.10 ± 8.30	8 weeks	“Blind selection”; sequence generation and allocation concealment not described.	See **Appendix S3** for details
Xia T, 2019 (16)	40 (20/20)	58.20 ± 4.00/56.50 ± 3.80	6 months	Random allocation reported; sequence generation and allocation concealment not described.	See **Appendix S3** for details
Zhang B, 2020 (17)	169 (84/85)	59.23 ± 0.23/58.20 ± 0.20	1 month	Allocation method not reported.	See **Appendix S3** for details
Lin LZ and Ji H, 2023 (18)	110 (55/55)	60.02 ± 3.28/60.34 ± 3.19	12 weeks	Random allocation reported; sequence generation and allocation concealment not described.	See **Appendix S3** for details

GQD, Gegen Qinlian Decoction; I/C, intervention group/control group; HbA1c, glycated hemoglobin; FPG, fasting plasma glucose; 2hPG, 2-hour postprandial glucose. All included studies were conducted in China and reported HbA1c, FPG, and 2hPG. NR, not reported.

### Risk of bias assessment

The risk-of-bias assessment is presented in **Figures 1** and **2**, with domain-specific judgments and supporting reasons provided in **Appendix S4**. Overall, all included trials were judged as having high risk of bias.

For bias arising from the randomization process, S1–S3 reported the use of random-number tables and therefore provided clearer evidence of randomization than the remaining trials. However, allocation concealment was not described. For S4–S8, the method of random sequence generation was unclear or insufficiently reported, which raised concerns about selection bias. Regarding deviations from intended interventions, none of the included trials used blinding or placebo control. Because GQD has distinctive sensory characteristics and the control groups received metformin alone, lack of blinding may have influenced co-interventions, adherence, or outcome-related behavior, even though the glycemic outcomes themselves were laboratory-based measures.

For missing outcome data, most trials did not provide sufficient information on attrition, exclusions after randomization, or whether analyses followed the intention-to-treat principle. Outcome measurement was considered less likely to introduce major bias because HbA1c, FPG, and 2hPG are objective laboratory indicators; however, details of laboratory procedures and assessor blinding were generally not reported. For selection of the reported result, no included trial had an accessible prospective protocol or trial registration record, which raised concerns about selective outcome reporting. These limitations contributed to the overall high risk-of-bias judgments and were considered in the GRADE downgrading.

### Certainty of evidence

The certainty of evidence for the three main glycemic outcomes was assessed using the GRADE framework and is summarized in **Table 2**. Because the included studies were randomized controlled trials, the initial certainty of evidence was rated as high. However, the certainty was downgraded for all three outcomes because of important methodological limitations in the included trials, substantial to extreme heterogeneity, and imprecision related to the small number of trials and participants included in the primary change-score analysis.

**Table 2:** Summary of findings (GRADE) for GQD plus metformin versus metformin monotherapy for T2DM based on the primary change-score analysis.

Outcomes	Follow-up	N participants (studies)	Effect (MD, 95% CI)	(GRADE)	Plain-language summary
HbA1c (%)	2–8 weeks	196 (2 RCTs)	-1.92 (-4.43 to 0.59)	Very low	The effect of GQD plus metformin on HbA1c is uncertain.
FPG (mmol/L)	2–8 weeks	196 (2 RCTs)	-1.08 (-1.71 to -0.44)	Very low	GQD plus metformin may reduce FPG, but the evidence is very uncertain.
2hPG (mmol/L)	2–8 weeks	196 (2 RCTs)	-1.73 (-3.05 to -0.42)	Very low	GQD plus metformin may reduce 2hPG, but the evidence is very uncertain.

HbA1c, glycated hemoglobin; FPG, fasting plasma glucose; 2hPG, 2-hour postprandial glucose; MD, mean difference; CI, confidence interval; GQD, Gegen Qinlian Decoction; T2DM, type 2 diabetes mellitus; RCTs, randomized controlled trials; GRADE, Grading of Recommendations Assessment, Development and Evaluation.

The certainty of evidence was downgraded for risk of bias because of high overall risk-of-bias judgments across the included trials, mainly related to insufficient reporting of randomization, lack of blinding or placebo control, and absence of prospectively registered protocols. The certainty was also downgraded for inconsistency because substantial to extreme heterogeneity was observed across the primary change-score analyses, and for imprecision because the analyses included only two trials with a limited number of participants. For HbA1c, the confidence interval crossed the line of no effect. Publication bias could not be formally assessed because fewer than ten studies were included.

Overall, the certainty of evidence was rated as very low for HbA1c, FPG, and 2hPG. For HbA1c, the evidence was downgraded because of high risk of bias, extreme inconsistency, and imprecision, as the confidence interval crossed the line of no effect. For FPG and 2hPG, although the pooled estimates favored GQD plus metformin, the certainty was also rated as very low because the results were based on only two trials and were affected by substantial heterogeneity and methodological limitations. Therefore, the GRADE assessment indicates that the true effects may be substantially different from the pooled estimates.

### Primary analysis of change-from-baseline values

**Figure 4:**
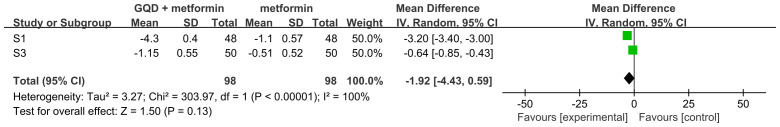
Forest plot for HbA1c (change scores; r = 0.50). Meta-analysis of changes from baseline in glycated hemoglobin levels between the combination therapy group and the metformin monotherapy group, assuming a correlation coefficient (r) of 0.50.

**Figure 5:**
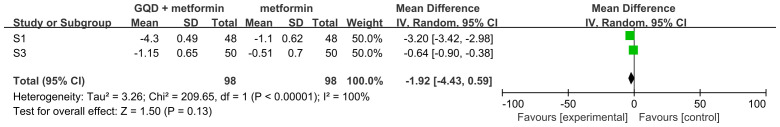
Forest plot for HbA1c (change scores; r = 0.25).Sensitivity analysis of HbA1c change scores using a conservative correlation coefficient (r) of 0.25 to assess the robustness of the primary outcome.

**Figure 6:**
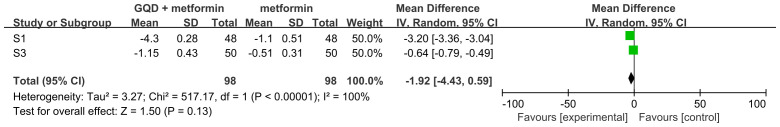
Forest plot for HbA1c (change scores; r = 0.75). Sensitivity analysis of HbA1c change scores using a liberal correlation coefficient (r) of 0.75 to evaluate the stability of the pooled effect estimate.

The primary analysis was based on change-from-baseline values. Only two trials (S1 and S3; 196 participants) provided sufficient baseline and post-treatment data for this analysis. Because the SDs of change scores were not directly reported, they were estimated using a prespecified correlation coefficient of r = 0.50 (**Figure 4**), with additional sensitivity checks using r = 0.25 and r = 0.75 (**Figures 5**, **6**).

For the primary outcome, GQD plus metformin did not show a statistically significant additional reduction in HbA1c compared with metformin alone in the change-score analysis (MD = -1.92, 95% CI -4.43 to 0.59; P = 0.13). This result remained statistically non-significant under the alternative correlation assumptions. Heterogeneity was extreme across the HbA1c change-score analyses (I² = 99%–100%), indicating substantial instability in the pooled estimate. Therefore, the primary analysis did not provide sufficient evidence to confirm a reliable additional effect of GQD on HbA1c.

For the secondary outcomes, the change-score analyses favored GQD plus metformin. Statistically significant reductions were observed for FPG (MD = -1.08, 95% CI -1.71 to -0.44; P = 0.0009) and 2hPG (MD = -1.73, 95% CI -3.05 to -0.42; P = 0.010). These findings were consistent across the tested correlation coefficients. However, heterogeneity remained substantial to extreme (I² = 85%–96%), and the analyses were based on a small number of trials. Accordingly, the FPG and 2hPG findings were interpreted as suggestive rather than confirmatory evidence.

### Sensitivity analysis 1 (post-treatment values; S1-S3)

This sensitivity analysis was restricted to the three trials with clearly reported randomization methods (S1–S3) and was based on post-treatment final values. Compared with metformin alone, GQD plus metformin showed statistically significant reductions in HbA1c (MD = -1.73, 95% CI -3.43 to -0.03; P = 0.05; **Supplementary Figure 1**), FPG (MD = -1.22, 95% CI -1.58 to -0.86; P < 0.00001; **Supplementary Figure 2**), and 2hPG (MD = -1.69, 95% CI -2.77 to -0.60; P = 0.002; **Supplementary Figure 3**). However, heterogeneity remained substantial to extreme across these analyses (I² = 87%–100%). Because this analysis was based on post-treatment values rather than change-from-baseline values, and because none of the included trials used blinding or placebo control, these findings were interpreted as supportive sensitivity evidence rather than confirmatory evidence.

### Sensitivity analysis 2 (post-treatment values; S1-S8)

When all eight trials were pooled using post-treatment final values, the effect estimates favored GQD plus metformin for all three glycemic outcomes. The pooled MDs were -1.21 for HbA1c (95% CI -1.99 to -0.43; P = 0.002; **Supplementary Figure 4**), -1.34 for FPG (95% CI -1.66 to -1.02; P < 0.00001; **Supplementary Figure 5**), and -2.01 for 2hPG (95% CI -2.71 to -1.31; P < 0.00001; **Supplementary Figure 6**). Nevertheless, substantial heterogeneity persisted across the all-study analyses (I² = 91%–99%). In addition, five of the eight trials did not clearly report the method of random sequence generation, and none used blinding or placebo control. Therefore, although the post-treatment sensitivity analyses showed statistically significant results, these findings should be interpreted cautiously and were not considered sufficient to override the more conservative change-score primary analysis.

### Safety reporting

Safety outcomes were incompletely and inconsistently reported across the included trials. Some studies mentioned adverse reactions or hypoglycemic episodes, but the definitions, timing, severity, causality assessment, and denominators for safety events were not consistently provided. No trial provided sufficient standardized safety data to support a reliable quantitative synthesis. Therefore, safety outcomes were summarized descriptively only. Overall, the available evidence was insufficient to determine whether adding GQD to metformin changes the risk of adverse events compared with metformin alone.

## Discussion

This systematic review and meta-analysis evaluated whether GQD provides additional glycemic benefit when used as an adjunct to metformin in adults with type 2 diabetes mellitus. To maintain a focused clinical question, we restricted the review to randomized controlled trials comparing GQD plus metformin with metformin alone and excluded studies involving additional hypoglycemic drugs. Overall, the findings suggest possible improvements in some glycemic parameters, particularly FPG and 2hPG, but the certainty of evidence remains very low because of methodological limitations, substantial heterogeneity, and imprecision.

The primary analysis was based on change-from-baseline values and was limited to two trials with sufficient baseline and post-treatment data. In this analysis, GQD plus metformin did not show a statistically significant additional reduction in HbA1c compared with metformin alone (MD = -1.92, 95% CI -4.43 to 0.59; P = 0.13). This non-significant result should not be interpreted as definitive evidence of no clinically important effect. The point estimate suggested a potentially meaningful reduction, but the confidence interval was wide and crossed the null value, indicating substantial imprecision. In addition, the heterogeneity was extreme, which further limited the interpretability of the pooled estimate. Therefore, the current evidence is insufficient to determine whether GQD plus metformin produces a reliable additional reduction in HbA1c.

The findings for FPG and 2hPG differed from those for HbA1c. In the primary change-score analyses, GQD plus metformin was associated with statistically significant reductions in FPG and 2hPG, suggesting a possible short-term glycemic signal. However, these results should be interpreted cautiously because they were based on only two trials and were affected by substantial heterogeneity. Thus, the FPG and 2hPG findings should be viewed as exploratory rather than confirmatory.

The discrepancy between the change-score analysis and the post-treatment sensitivity analyses also requires cautious interpretation. The post-treatment analyses, including the analysis of all eight trials, favored GQD plus metformin across HbA1c, FPG, and 2hPG. However, post-treatment final values are more vulnerable to baseline imbalance than change-from-baseline values, especially when randomization procedures are insufficiently reported. Therefore, the statistically significant post-treatment findings were considered supportive sensitivity evidence, but they were not used to override the more conservative primary change-score analysis.

The interpretation of HbA1c in short-duration trials also deserves attention. HbA1c reflects average glycemia over approximately the preceding 2–3 months and therefore changes in HbA1c may be less responsive in trials with treatment durations shorter than 12 weeks (19). Several included trials had relatively short follow-up periods, including trials lasting 2 to 8 weeks. We retained these studies because HbA1c was reported as a prespecified glycemic outcome and because excluding them would further reduce the already limited evidence base. Nevertheless, the short duration of several trials may have reduced the ability to detect sustained changes in HbA1c and contributed to inconsistency across studies. This issue was considered when interpreting the HbA1c findings and when downgrading the certainty of evidence.

In addition to the statistical findings, the high risk of bias in the included trials should also be considered. Effective blinding in trials of traditional Chinese medicine decoctions remains challenging. The distinctive sensory characteristics of GQD, including its pungent aroma, strong bitterness, and dark color, make the implementation of a true double-blind design difficult when the control group receives metformin alone. Accordingly, the certainty of evidence was downgraded to very low within the GRADE assessment. This downgrading does not diminish the value of HbA1c as a measure of long-term glycemic control; rather, it reflects the methodological limitations and statistical instability of the available evidence. Future placebo formulations may use flavoring agents, coloring agents, bittering agents, and water to better simulate the sensory characteristics of GQD and facilitate blinding (20, 21).

Safety reporting was insufficient and inconsistent across the included trials. Several studies either did not report adverse events in detail or reported them only briefly without standardized definitions, grading criteria, or attribution to treatment. Because the timing, severity, and causality of adverse events were generally unclear, a reliable quantitative synthesis of safety outcomes was not feasible. This limitation is clinically important because any potential glycemic benefit of GQD as an adjunctive therapy must be weighed against its safety profile.

Mechanistic findings should also be interpreted cautiously. Some experimental and clinical studies have suggested that GQD may influence glucose metabolism through pathways related to gut microbiota, inflammation, oxidative stress, or insulin resistance (5, 22). However, the present meta-analysis was designed to evaluate clinical glycemic outcomes rather than mechanisms, and the included trials provided limited mechanistic data. Therefore, mechanistic hypotheses should be considered supportive background information rather than direct evidence of clinical efficacy.

Several limitations of the review process should also be acknowledged. First, this review was not prospectively registered, and no publicly available protocol was prepared before the review was conducted. Although the eligibility criteria and planned outcomes were defined before data extraction and analysis, the absence of prospective registration may limit transparency and increase the possibility of selective methodological decisions. Second, the certainty of evidence was limited by the methodological weaknesses of the included trials, substantial heterogeneity, and imprecision of the pooled estimates. According to the GRADE assessment, the certainty of evidence for the main outcomes was rated as very low, indicating that the true effect may be substantially different from the pooled estimates. Therefore, the statistically significant findings for FPG and 2hPG should be interpreted as exploratory rather than confirmatory. Third, all included trials were conducted in China, and most were published in Chinese-language journals. This may limit the generalizability of the findings to other healthcare settings, populations, dietary backgrounds, and regulatory environments for herbal medicine. Differences in the quality control, preparation, and clinical use of GQD across regions may also affect the applicability of the pooled results outside the original study settings.

Substantial to extreme heterogeneity was observed across the meta-analyses. This heterogeneity was likely multifactorial. Potential sources included differences in treatment duration, sample size, baseline glycemic status, GQD formulation and dosage, metformin dose, reporting quality, and risk-of-bias profile. We considered exploring heterogeneity through subgroup analyses according to treatment duration, GQD regimen, and randomization reporting; however, these analyses were not considered reliable because only a small number of trials were available for each outcome, and the primary change-score analysis included only two trials. Meta-regression was also not appropriate because of the limited number of included studies. Therefore, heterogeneity was addressed descriptively and through sensitivity analyses rather than through formal subgroup modelling.

The possibility of publication bias should also be considered. Fewer than ten trials were included, so funnel-plot assessment or statistical tests for small-study effects were not appropriate. In addition, most included trials were small, single-center studies published in Chinese journals, and none had an accessible prospective protocol or trial registration record. These factors increase the possibility that studies with favorable results were more likely to be published or reported. Accordingly, the pooled estimates should be interpreted cautiously.

## Conclusion

Current evidence is insufficient to establish GQD as a definitively superior adjunctive treatment to metformin for type 2 diabetes mellitus. Although the post-treatment sensitivity analyses favored GQD plus metformin across HbA1c, FPG, and 2hPG, the primary change-score analysis did not confirm a statistically significant additional reduction in HbA1c. The observed reductions in FPG and 2hPG suggest a possible glycemic benefit, but these findings should be interpreted as exploratory because of substantial heterogeneity, high risk of bias, limited reporting quality, and the absence of blinding or placebo control in most included trials. Therefore, GQD may have potential as an adjunctive therapy, but its routine clinical use cannot yet be supported because high-certainty evidence is lacking. Future studies should be prospectively registered, adequately powered, placebo-controlled where feasible, and reported transparently according to CONSORT and related reporting standards.

## Data Availability

The original contributions presented in the study are included in the article/**Supplementary Material**. Further inquiries can be directed to the corresponding author.

## Supplementary material

Supplementary Figure 1 Forest plot for HbA1c based on post-treatment values in trials with clearly reported randomization methods (S1–S3).

Supplementary Figure 2 Forest plot for FPG based on post-treatment values in trials with clearly reported randomization methods (S1–S3).

Supplementary Figure 3 Forest plot for 2hPG based on post-treatment values in trials with clearly reported randomization methods (S1–S3).

Supplementary Figure 4 Forest plot for HbA1c based on post-treatment values in all included trials (S1–S8).

Supplementary Figure 5 Forest plot for FPG based on post-treatment values in all included trials (S1–S8).

Supplementary Figure 6 Forest plot for 2hPG based on post-treatment values in all included trials (S1–S8).
