# Supplementary figures and images for: Efficacy of Gegen Qinlian decoction plus metformin for type 2 diabetes mellitus: a systematic review and meta-analysis of randomized controlled trials
Source: Front Endocrinol (Lausanne). 2026 Jul 17;17:1837588. doi: 10.3389/fendo.2026.1837588 (PMC13423648; doi:10.3389/fendo.2026.1837588)

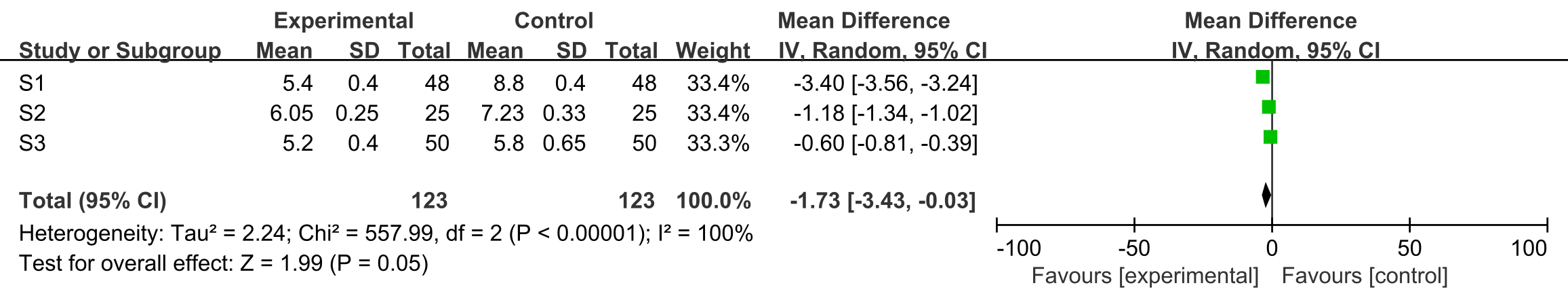

Supplement: Supplementary Figure 1 — Forest plot for HbA1c based on post-treatment values in trials with clearly reported randomization methods (S1–S3). [file Image1.tiff]

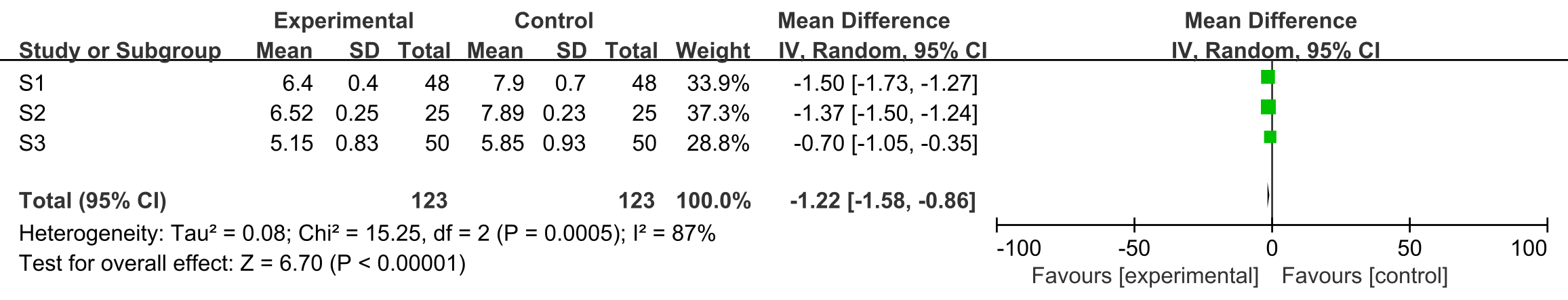

Supplement: Supplementary Figure 2 — Forest plot for FPG based on post-treatment values in trials with clearly reported randomization methods (S1–S3). [file Image2.tiff]

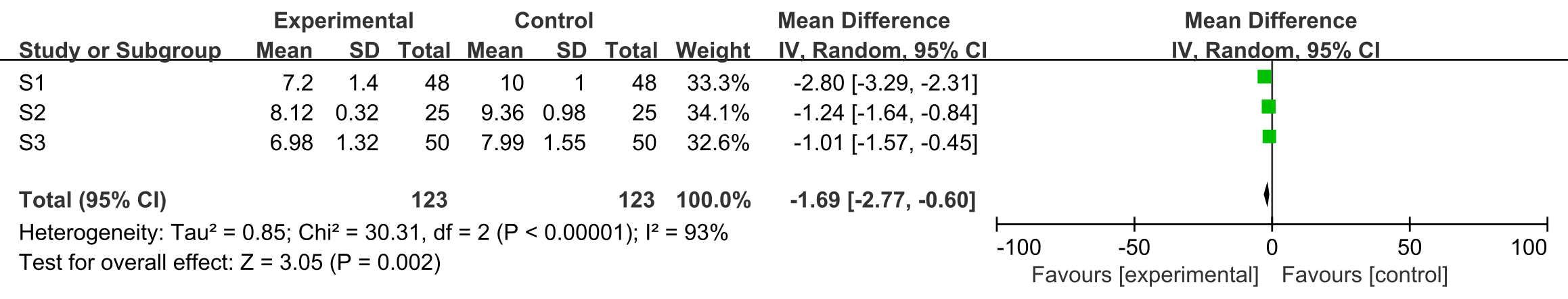

Supplement: Supplementary Figure 3 — Forest plot for 2hPG based on post-treatment values in trials with clearly reported randomization methods (S1–S3). [file Image3.tiff]

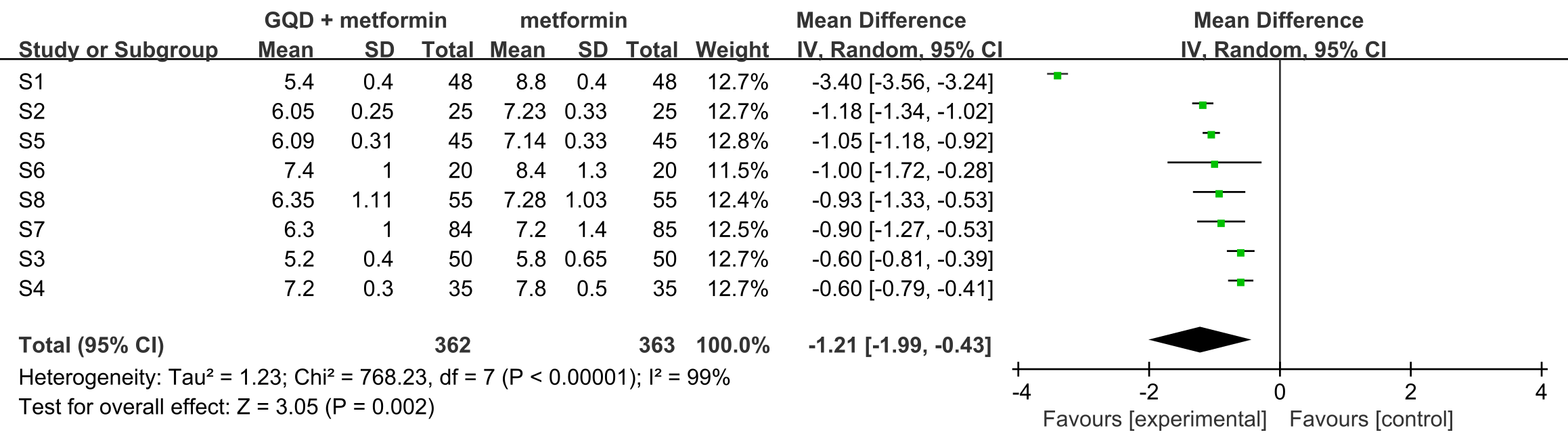

Supplement: Supplementary Figure 4 — Forest plot for HbA1c based on post-treatment values in all included trials (S1–S8). [file Image4.tiff]

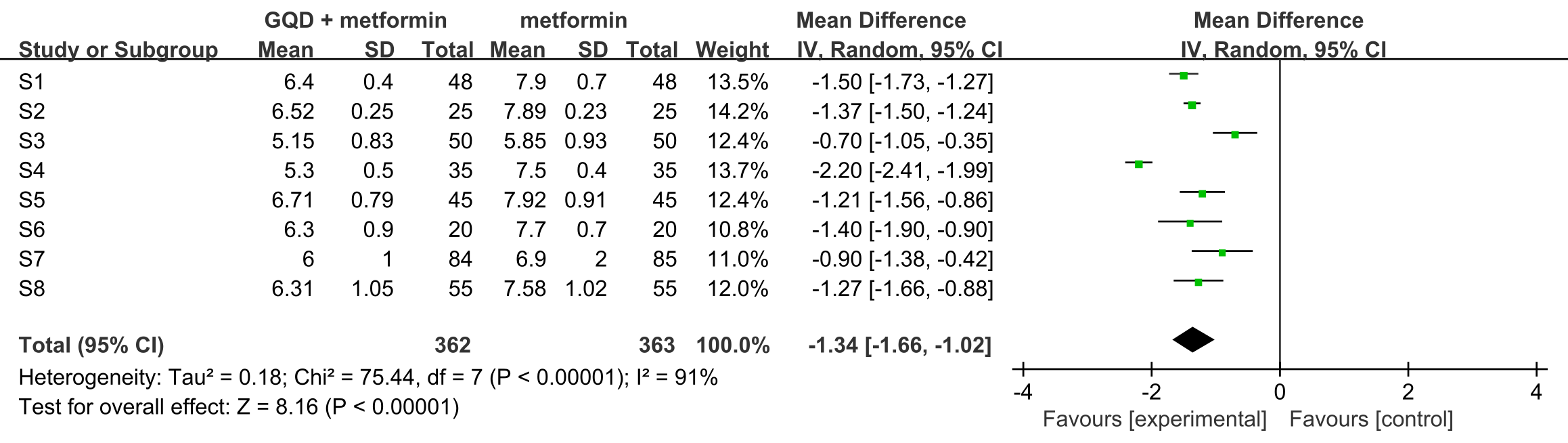

Supplement: Supplementary Figure 5 — Forest plot for FPG based on post-treatment values in all included trials (S1–S8). [file Image5.tiff]

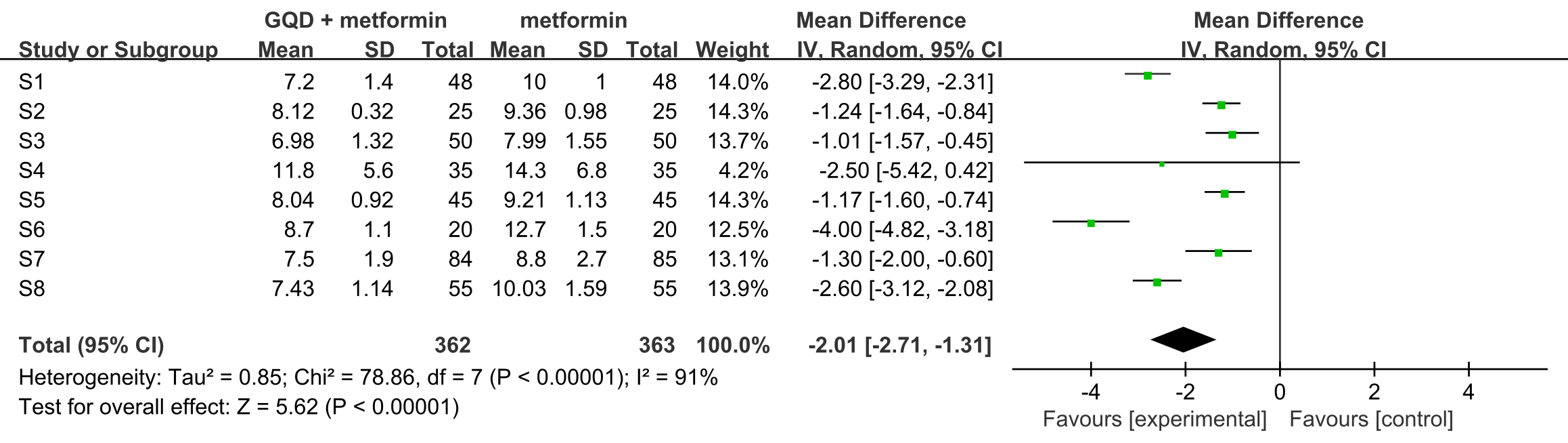

Supplement: Supplementary Figure 6 — Forest plot for 2hPG based on post-treatment values in all included trials (S1–S8). [file Image6.tiff]
