# Supplementary material for: Efficacy of Gegen Qinlian decoction plus metformin for type 2 diabetes mellitus: a systematic review and meta-analysis of randomized controlled trials
Source: Front Endocrinol (Lausanne). 2026 Jul 17;17:1837588. doi: 10.3389/fendo.2026.1837588 (PMC13423648; doi:10.3389/fendo.2026.1837588)
Supplement: Supplementary file 8 [file SupplementaryFile2.docx]

**Appendix S2. Detailed search strategies for all electronic databases.**

All databases were last searched on 31 January 2026. Record counts are reported before duplicate removal.

| Database | Records | Search strategy |
| --- | --- | --- |
| CNKI | 161 | 篇关摘: (葛根芩连汤 + 复方葛根芩连饮 + 葛根芩连) AND 篇关摘: (2型糖尿病 + 2型DM + T2DM + 糖尿病) AND 篇关摘: (随机 + 随机对照 + 随机分组 + 随机对照试验) |
| Wanfang Data | 228 | 主题:(葛根芩连汤 OR 复方葛根芩连饮 OR 葛根芩连) AND 主题:(2型糖尿病 OR 2型DM OR 糖尿病) AND 主题:(随机 OR 随机对照 OR 随机化分配) |
| PubMed | 9 | ("Gegen Qinlian"[tiab] OR "Gegen Qinlian decoction"[tiab] OR "Ge-Gen-Qin-Lian"[tiab] OR Gegenqinlian[tiab]) AND ("Diabetes Mellitus, Type 2"[Mesh] OR "type 2 diabetes"[tiab] OR T2DM[tiab]) AND (randomized[tiab] OR randomised[tiab] OR trial[tiab] OR "randomized controlled trial"[pt]) NOT (meta-analysis[pt] OR systematic review[pt]) |
| Embase | 21 | ('gegen qinlian':ti,ab OR 'gegen qinlian decoction':ti,ab OR 'ge gen qin lian':ti,ab) AND ('type 2 diabetes mellitus'/exp OR 'type 2 diabetes':ti,ab OR T2DM:ti,ab) AND (random*:ti,ab OR 'randomized controlled trial'/exp) |
| Web of Science | 1 | TS=("Gegen Qinlian decoction" OR "Gegen Qinlian" OR "Ge-Gen-Qin-Lian" OR Gegenqinlian OR GQD) AND TS=("type 2 diabetes" OR T2DM OR "type II diabetes" OR "Diabetes Mellitus, Type 2") AND TS=(random* OR randomised OR randomized OR trial*) |
| CENTRAL | 0 | ("Gegen Qinlian" OR "Gegen Qinlian decoction" OR "Ge-Gen-Qin-Lian" OR Gegenqinlian OR GQD) AND ("type 2 diabetes" OR T2DM OR "type II diabetes" OR "Diabetes Mellitus, Type 2") |
