# Supplementary material for: Efficacy of Gegen Qinlian decoction plus metformin for type 2 diabetes mellitus: a systematic review and meta-analysis of randomized controlled trials
Source: Front Endocrinol (Lausanne). 2026 Jul 17;17:1837588. doi: 10.3389/fendo.2026.1837588 (PMC13423648; doi:10.3389/fendo.2026.1837588)
Supplement: Supplementary file 9 [file SupplementaryFile3.docx]

Appendix S3. Detailed GQD and metformin regimens in the included randomized controlled trials.

| **Study ID** | **GQD composition** | **GQD administration and modifications** | **Metformin regimen in intervention and control groups** |
| --- | --- | --- | --- |
| S1 | Compound GQD: Gegen 20 g, Huanglian 12 g, Huangqin 9 g, and Gancao 6 g. | One dose daily; decocted and taken orally twice daily. Individual modifications were permitted according to symptoms. | Metformin 500 mg/day in both groups. |
| S2 | GQD: Gegen 60 g, Huangqin 22.5 g, Huanglian 22.5 g, Ganjiang 3.5 g, and Shenggancao 15 g. | One dose daily; decocted and taken orally twice daily. Individual modifications were permitted according to symptoms. | Metformin hydrochloride 0.25 g per dose, orally three times daily after meals, in both groups. |
| S3 | GQD: Gegen 60 g, Huangqin 22.5 g, Huanglian 22.5 g, Shenggancao 15 g, and Ganjiang 3.5 g. | One dose daily; decocted to approximately 200 mL and taken orally twice daily. | Metformin hydrochloride 0.25 g per dose, orally three times daily, in both groups. |
| S4 | GQD: Gegen 20 g, Huanglian 5 g, Huangqin 20 g, and Gancao 5 g. | One dose daily; decocted to approximately 300 mL and taken orally twice daily, in the morning and evening. | Initial metformin dose 0.25 g per dose, orally twice daily; adjusted according to glucose monitoring to a maximum daily dose of 2.0 g, in both groups. |
| S5 | GQD decoction pieces: Gegen 60 g, Huangqin 22.5 g, Huanglian 22.5 g, Shenggancao 15 g, and Ganjiang 3.5 g. | One dose daily; decocted and taken orally twice daily. | Metformin hydrochloride 0.25 g per dose, orally three times daily during or after meals, in both groups. |
| S6 | GQD: Gegen 60 g, Huangqin 22.5 g, Huanglian 22.5 g, Ganjiang 3.5 g, and Shenggancao 15 g. | One dose daily. Pingwei San, Mianyinchen, or Liuyi San could be added according to syndrome differentiation. | Metformin 0.25–0.50 g per dose, orally three times daily, in both groups. |
| S7 | GQD: Gegen 30 g, Huangqin 20 g, Huanglian 6 g, and Zhigancao 6 g. | One dose daily; decocted and taken orally twice daily, in the morning and evening. | Metformin hydrochloride 0.25 g per dose, orally three times daily, in both groups. |
| S8 | GQD: Gegen 30 g, Huanglian 12 g, Huangqin 9 g, and Zhigancao 6 g. | One dose daily; decocted to approximately 400 mL and taken orally twice daily, in the morning and evening. | Initial metformin dose 0.5 g per dose, orally twice daily with meals; increased when necessary to 1.0 g per dose, orally three times daily, in both groups. |

Abbreviations: GQD, Gegen Qinlian Decoction; Gegen, Puerariae Lobatae Radix; Huangqin, Scutellariae Radix; Huanglian, Coptidis Rhizoma; Ganjiang, Zingiberis Rhizoma; Shenggancao/Zhigancao/Gancao, Glycyrrhizae Radix; oral, administration by mouth.

Note: The intervention group received GQD in addition to the metformin regimen described above, whereas the control group received the same metformin regimen alone. Treatment duration is reported in Table 1.
