# Supplementary material for: Efficacy of Gegen Qinlian decoction plus metformin for type 2 diabetes mellitus: a systematic review and meta-analysis of randomized controlled trials
Source: Front Endocrinol (Lausanne). 2026 Jul 17;17:1837588. doi: 10.3389/fendo.2026.1837588 (PMC13423648; doi:10.3389/fendo.2026.1837588)
Supplement: Supplementary file 10 [file SupplementaryFile4.docx]

**Appendix S4. Domain-specific RoB 2 judgments for the included randomized controlled trials.**

Judgments were made for the main glycemic outcomes (HbA1c, FPG, and 2hPG). D1, bias arising from the randomization process; D2, bias due to deviations from intended interventions; D3, bias due to missing outcome data; D4, bias in measurement of the outcome; D5, bias in selection of the reported result.

| **Study ID** | **D1** | **D2** | **D3** | **D4** | **D5** | **Overall** | **Support for judgment** |
| --- | --- | --- | --- | --- | --- | --- | --- |
| S1 Li H, 2018 | Some concerns | Some concerns | Some concerns | Low risk | Some concerns | High risk | Random allocation was reported; the retained extraction record specified use of a random-number table. Allocation concealment was not reported. No placebo or blinding was reported. Information on adherence, co-interventions, missing outcome data, post-randomization exclusions, and the analysis population was insufficiently reported. HbA1c, FPG, and 2hPG were objective laboratory outcomes. No protocol or prespecified statistical analysis plan was reported in the article. |
| S2 Li WJ and Li M, 2021 | Some concerns | Some concerns | Some concerns | Low risk | Some concerns | High risk | Random allocation was reported; the retained extraction record specified use of a random-number table. Allocation concealment was not reported. No placebo or blinding was reported. Information on adherence, co-interventions, missing outcome data, post-randomization exclusions, and the analysis population was insufficiently reported. HbA1c, FPG, and 2hPG were objective laboratory outcomes. No protocol or prespecified statistical analysis plan was reported in the article. |
| S3 Xiong QJ, 2019 | Some concerns | Some concerns | Some concerns | Low risk | Some concerns | High risk | Random allocation was reported; the retained extraction record specified use of a random-number table. Allocation concealment was not reported. No placebo or blinding was reported. Information on adherence, co-interventions, missing outcome data, post-randomization exclusions, and the analysis population was insufficiently reported. HbA1c, FPG, and 2hPG were objective laboratory outcomes. No protocol or prespecified statistical analysis plan was reported in the article. |
| S4 Zhang HF, 2019 | Some concerns | Some concerns | Some concerns | Low risk | Some concerns | High risk | Seventy participants were reported to have been randomly and equally divided into two groups of 35; however, the method of random-sequence generation and allocation concealment was not described. No placebo or blinding was reported. Information on adherence, co-interventions, missing outcome data, post-randomization exclusions, and the analysis population was insufficiently reported. HbA1c, FPG, and 2hPG were objective laboratory outcomes. No protocol or prespecified statistical analysis plan was reported in the article. |
| S5 Pang XY et al., 2018 | Some concerns | Some concerns | Some concerns | Low risk | Some concerns | High risk | Participants were allocated by “blind selection”; however, the report did not describe a reproducible random-sequence generation method or allocation concealment. No placebo or blinding was reported. Information on adherence, co-interventions, missing outcome data, post-randomization exclusions, and the analysis population was insufficiently reported. HbA1c, FPG, and 2hPG were objective laboratory outcomes. No protocol or prespecified statistical analysis plan was reported in the article. |
| S6 Xia T, 2019 | Some concerns | Some concerns | Some concerns | Low risk | Some concerns | High risk | Participants were reported to have been randomly divided into two groups, but the method of random-sequence generation and allocation concealment was not described. No placebo or blinding was reported. Information on adherence, co-interventions, missing outcome data, post-randomization exclusions, and the analysis population was insufficiently reported. HbA1c, FPG, and 2hPG were objective laboratory outcomes. No protocol or prespecified statistical analysis plan was reported in the article. |
| S7 Zhang B, 2020 | Some concerns | Some concerns | Some concerns | Low risk | Some concerns | High risk | Both groups received metformin hydrochloride tablets, and the intervention group additionally received GQD. The treatment duration was 1 month. The report did not describe the random allocation method or allocation concealment. No placebo or blinding was reported. Information on adherence, co-interventions, missing outcome data, post-randomization exclusions, and the analysis population was insufficiently reported. HbA1c, FPG, and 2hPG were objective laboratory outcomes. No protocol or prespecified statistical analysis plan was reported in the article. |
| S8 Lin LZ and Ji H, 2023 | Some concerns | Some concerns | Some concerns | Low risk | Some concerns | High risk | Participants were reported to have been randomly divided into two groups, but the method of random-sequence generation and allocation concealment was not described. No placebo or blinding was reported. Information on adherence, co-interventions, missing outcome data, post-randomization exclusions, and the analysis population was insufficiently reported. HbA1c, FPG, and 2hPG were objective laboratory outcomes. No protocol or prespecified statistical analysis plan was reported in the article. |

Notes: Judgments were made for the effect of assignment to intervention on the main glycemic outcomes (HbA1c, FPG, and 2hPG). D1, bias arising from the randomization process; D2, bias due to deviations from intended interventions; D3, bias due to missing outcome data; D4, bias in measurement of the outcome; D5, bias in selection of the reported result.

D1 was judged as having some concerns because allocation concealment was not reported in any study, and several studies did not describe a reproducible random-sequence generation method. D2 was judged as having some concerns because no study reported placebo control or participant/personnel blinding, and information on adherence, co-interventions, and deviations from intended interventions was limited. D3 was judged as having some concerns because information on missing outcome data, post-randomization exclusions, and the analysis population was insufficiently reported. D4 was judged as low risk because the prespecified glycemic outcomes were objective laboratory measures and no evidence suggested differential outcome measurement between groups. D5 was judged as having some concerns because no protocol or prespecified statistical analysis plan was reported in the included articles.

Overall risk of bias was judged as high because multiple domains raised some concerns that substantially reduced confidence in the reported effects.
